# Risk Scores for Patients with Chest Pain: Evaluation in the Emergency Department

**DOI:** 10.2174/157340311795677662

**Published:** 2011-02

**Authors:** B.E Backus, A.J Six, J.H Kelder, W.B Gibler, F.L Moll, P.A Doevendans

**Affiliations:** 1 Unversitair Medisch Centrum Utrecht, The Netherlands; 2 Hofpoort Ziekenhuis Woerden, The Netherlands; 3 St Antonius Ziekenhuis Nieuwegein, The Netherlands; 4 University of Cincinnati, Cincinnati, Ohio, USA

**Keywords:** Chest pain, risk scores, emergency department.

## Abstract

Chest pain is a common reason for presentation to the emergency department (ED). Absolute criteria for Acute Coronary Syndrome without ST elevation (NSTE-ACS) are lacking. An acute coronary syndrome (ACS) needs to be distinguished from a variety of other cardiac and non-cardiac diseases that may cause chest pain.

For patients with confirmed ACS, several scoring methods can be applied in order to distinguish patients in the coronary care unit who may benefit most from therapies. The PURSUIT, TIMI, GRACE and FRISC risk scores are well validated with this respect. However, none of these risk scores has been used in the identification of an ACS in the emergency setting. The vast majority of patients with chest pain due to causes other than ACS were not evaluated in these trials. An evidence-based systematic stratification and policy for these patients does not currently exist.

The more recently developed HEART score is specifically designed to stratify all chest pain patients in the ED. The HEART score was validated in a retrospective multicenter study and proved to be a strong predictor of event free survival on one hand and potentially life threatening cardiac events on the other hand. The HEART score facilitates risk stratification of chest pain patients in the ED.

## INTRODUCTION

Chest pain is one of the most common reasons for patients to present to the emergency department (ED). An acute coronary syndrome (ACS) needs to be distinguished from a variety of other cardiac and non cardiac diseases that may cause chest pain. In a number of cases, a diagnosis can be made quickly, in particular in case of ST-segment elevation acute myocardial infarction (STEMI). However, STEMI patients represent only a small percentage of all chest pain patients in this setting. A variety of other diseases may mimic ACS, such as pleural and pericardial irritations, gastro-intestinal reflux, pulmonary embolism, hyperventilation, musculoskeletal pain and cholecystitis [1-3]. 

The challenge in the ED is not only to identify patients at the highest risk, but also to identify patients with non-urgent diseases or even the absence of disease. These patients may be discharged immediately with minimal testing or intervention. Clearly, when treated as ACS, the latter will be prone to unnecessary risks of various treatments, including the side effects of medication or radiation. In addition, this causes the occupation of hospital beds through admission of such patients and associated increase in medical costs. With the population’s increasing age and advancing medical techniques, healthcare costs are a critical issue in many countries. Awareness of these costs as well as treatment risks is necessary before considering a certain strategy for the individual patient [2].

Regarding patients with ACS, the diagnosis is confirmed in the vast majority of cases where significant ECG changes such as STEMI and/or increased levels of myocardial markers in plasma are present. However, absence of such abnormalities doesn’t exclude ACS. Therefore, the diagnosis of ACS is felt to be difficult to exclude in the early stage of the diagnostic process. It is important to make the diagnosis quickly, as patients benefit significantly from early treatment [3]. With this perspective in mind and the possible life threatening character of ACS, guidelines for chest pain patients are mainly focussed on the identification of those patients at the highest risk of an ACS. High risk patients will benefit most from early aggressive therapies. With this approach, the current guidelines disregard the many chest pain patients with a wide selection of non-urgent diagnoses in whom admission is not necessary. 

### Risk Scores

Several scoring methods are developed in order to distinguish patients in the ED or coronary care unit at the highest risk of an ACS or an adverse outcome, who may benefit most from aggressive therapies.

## PURSUIT

The PURSUIT score (2000) was developed in a multinational randomized clinical trial (Platelet glycoprotein IIb/IIIa in Unstable angina: Receptor Suppression Using Integrilin (eptifibatide) Therapy), with 9,461 patients, comparing eptifibatide (Integrilin) to placebo in the management of Unstable Angina (UA) or NonST-elevation Myocardial Infarction (NSTEMI) [4]. By use of multivariate regression analysis the investigators identified seven risk predictors for death and myocardial infarction (MI) in patients with acute coronary syndromes. Five of these risk factors were then combined into a scoring system: higher age, sex, worst Canadian Cardiovascular Society (CCS) class of angina, signs of heart failure and ST-segment depression on the index ECG. The investigators did not include tachycardia and low systolic blood pressure in the final risk score (Table **I**). Scoring each of the five elements results in a possible score ranging from 1 to 18. The PURSUIT score predicts the risk of death or death/MI at 30 days after admission. According to the PURSUIT score ACS patients are divided into low, intermediate and high risk patients, with suggested therapies of early discharge, “watchful waiting” and aggressive antiplatelet / early invasive strategies respectively. The c-statistic of the original study for predicting the primary endpoint was 0.84 for death alone and 0.67 for the composite endpoint of death/MI. 

## TIMI

The TIMI risk score (2000) is derived from the Thrombolysis in Myocardial Infarction (TIMI)-11B trial, a multinational, randomized clinical trial, comparing unfractionated heparin to enoxaparin, which included all patients with confirmed ACS [5]. Data from 1,957 patients enrolled in the unfractionated heparin group were used to identify twelve elements of typical ACS findings by use of multivariate regression analysis. Seven of these elements remained statistically significant in a multivariate analysis. Together these seven elements compose the TIMI score for unstable angina/NSTEMI: age ≥ 65 years, ≥3 classical risk factors for coronary artery disease (CAD), known CAD, use of Aspirin in the past 7 days, severe angina in the past 24 hours, elevated cardiac markers and ST-deviation ≥ 0.5 mm (Table **II**). Each of these elements can be assigned with 0 or 1 points, resulting in a score of 0-7. The TIMI score predicts the risk of all cause mortality, MI and severe recurrent ischemia requiring urgent revascularization within 14 days after admission as well as benefit of enoxaparin. Event rates increased significantly with increasing TIMI-scores. According to the TIMI score patients are divided into low (score 0-2), intermediate (score 3-4) and high (score 5-7) risk categories. The c-statistic of the TIMI score in the original trial was 0.65. The TIMI score was validated internally and externally in the enoxaparin group of the TIMI 11B trial and in both groups of the ESSENCE trial [5]. This validation showed comparable results with a mean c-statistic of 0.63. In addition the TIMI score is validated in several other databases and was compared with other scoring systems [6-8]. In various succeeding trials the TIMI risk score was applied in analyzing treatment efficacy in various ACS risk groups. 

## GRACE

The GRACE score (2003) was developed in a multinational registry of 11,389 ACS patients (Global Registry of Acute Coronary Events) [9,10]. Registration was performed prospectively and retrospectively. Patients who died within 24 hour after admission were excluded. After data collection, by use of multivariate logistic regression analysis, the investigators identified eight independent risk factors for in-hospital death and post-discharge death at 6 months. These risk factors were then combined into a scoring system, consisting of hemodynamic, laboratory, ECG and patient specific findings: Killip class for congestive heart failure (CHF), systolic blood pressure at presentation (SBP), heart rate at presentation (HR), age, creatinine level, cardiac arrest at admission, ST-segment deviation on the index ECG and elevated cardiac enzyme levels. Each element has its own scoring, resulting in a possible score ranging from 1 to 372 (Table **III**). 

Event rates increased significantly with increasing GRACE-scores, ranging from ≤0.2% to ≥52% chance of in-hospital death. The investigators did not divide patients into different risk categories. However, the individual risk of in-hospital death may be used for optimal triage and management.

The c-statistic of the GRACE score in the original database was 0.83. The GRACE score was directly validated in a subsequent cohort of 3,972 patients and in 12,142 patients enrolled in the Global Utilization of Streptokinase and Tissue Plasminogen Activator for Occluded Coronary Arteries (GUSTO)-IIb trial [11]. This validation showed comparable results with c-statistic results of 0.84 and 0.79 respectively. The c-statistics were similar for patients with (0.83) and without (0.82) ST segment deviation, with (0.81) and without (0.83) elevated cardiac markers and for patients younger than 65 years (0.78) or older than 65 years of age (0.82).

In addition, the GRACE score has been validated in several other databases [9] and was compared with other scoring systems in various succeeding trials [6-8]. 

## FRISC

The FRISC score (2004) is based on the FRISC (Fast Revascularisation in Instability in Coronary disease) II trial [12]. A multicenter, randomized clinical trial, which included patients with unstable coronary artery disease. By use of multivariate regression analysis data from 1,235 patients enrolled in the non-invasive cohort were used to identify seven parameters as independent predictors of death/MI in patients with unstable angina. Together these seven parameters compose the FRISC score, consisting of age ≥ 70 years, male gender, diabetes, previous MI, ST-segment depression on admission, elevated levels of Troponin and elevated levels of Interleukin 6 or CRP (Table **IV**). Each of these elements can be appreciated with 0 or 1 points, resulting in a score of 0-7. The c-statistic of the FRISC score for the prediction of death was 0.77 and for death/MI 0.70. Using different age cut offs had only minimal effect on the accuracy. Use of CRP alone instead of CRP and Interleukin-6 decreased the c-statistics to 0.76 and 0.68 respectively.

Patients were categorized into low, intermediate and high risk, based on the FRISC scores of 0-2, 3-4 and 5-7. The FRISC score is the only risk score that focussed on the treatment effect of early invasive strategies in ACS. To evaluate this effect the developed risk score was also performed on the invasive cohort with 1,222 patients. In the high risk group mortality reduced from 15.4 – 5.2%, while the composite endpoint of death and MI was reduced in both intermediate and high risk groups. Therefore, investigators recommended early invasive strategies for patients with a FRISC score ≥ 3.

## HEART

Recently (2008), the HEART risk score was developed for chest pain patients presenting to the ED [13,14]. The composition of the HEART score was not based on multivariate regression analysis but on the decision making clinical factors according to expert opinion. The HEART score is composed of five parameters of clinical judgement: History, ECG, Age, Risk factors and Troponin. By appreciating each of these five elements with 0, 1 or 2 each patient patients will receive a score of 0-10 (Table **V**). 

The HEART score was consecutively validated in a single center retrospective study in 122 patients [13] and a multicenter retrospective investigation in 880 patients [14]. These studies were conducted in all patients presenting to the ED for chest pain within the first quarter of 2006. No other inclusion or exclusion criteria were used. The investigators calculated the predictive value of the HEART score for the combined endpoint of MI, Percutaneous Coronary Intervention (PCI), Coronary Artery Bypass Grafting (CABG) or death within 6 weeks after presentation. 

Event rates increased significantly with increasing HEART scores. The c-statistic was 0.90, indicating good to excellent discriminative power. The HEART score divides patients into low (0-3), intermediate (4-6) or high risk groups (7-10), with mean risks of an event of 0.9%, 12% and 65%, respectively. Consequently, an evidence-based decision may be made to discharge the patient from the ED or to admit for clinical observation or immediate aggressive therapies. 

In addition, the HEART score is currently being validated in a multicenter prospective study in 2440 patients at 10 hospitals. Follow up will be finalised by the end of the year 2010. 

### Clinical Practice

Chest pain patients in the ED create uncertainty for all treating physicians. The decision to discharge a patient where ACS cannot be excluded may result in a serious life-threatening outcome, while on the other hand, admission in case of atypical chest pain can lead to unnecessary medical treatment and costs. Risk score models may help the physician in making a timely decision in the emergency setting. In clinical practice, simple risk scores may be favourable, in particular when they can be calculated at the patient’s bedside.

For several years, researchers tried to develop a risk score for chest pain patients. Most of these risk scores have turned out to be difficult to use, require the use of a computer and, more importantly, are only validated for a selected group of patients such as STEMI or non-STEMI patients in the coronary care unit. The main purpose of all these risk scores was not to make a diagnosis, but to identify the subset of high risk ACS patients who are likely to benefit most from aggressive therapies. 

For NSTE-ACS, several risk scores have been developed and validated in large patient populations. Most useful are the PURSUIT [4], TIMI [5] and [6] GRACE [9,10] risk scores, which were compared by De Araújo Gonçalves6 and Yan [7]. Despite the firm scientific basis for all three scoring systems and the recommendations in the guidelines, none is widely applied in clinical practice. 

There is a clear difference in approach to patients admitted at the coronary care unit and patients presenting to the ED with suspected ACS. In “real life”, as experienced by every physician, the whole range of chest pain patients presenting to the ED runs from atypical chest pain to acute myocardial infarction. Therefore, the ideal risk score is capable of identifying patients at both ends of the spectrum.

### Applicability Per Score

The PURSUIT study was conducted before the general introduction of the troponin assay. This crucial test, which is now generally applied, was not included in the PURSUIT score. This is one of the reasons why the score has not found its place in routine clinical practice. Another objection is that the PURSUIT score is determined for more than 50% by the age of the patient. Not surprisingly, higher ages of patients accompany higher mortality rates. This knowledge does not help the clinician to make better decisions in the emergency setting. The PURSUIT score has good predictive power for death alone (c-statistic 0.84), but rather poor predictive power for the combined endpoint of death/MI (c-statistic 0.67).

The c-statistic of the TIMI score is 0.65 for the combined endpoint, indicating poor predictive power, but its simplicity makes it more useful than other scores. Even though the TIMI score is simple to calculate, it allows only binary choices, thereby ignoring the fact that many variables have “grey levels”.

The GRACE risk score has a good discriminative power with a c-statistic of 0.83. However, the complexity of the system requires special calculating tools to estimate risk at the bedside. Like the PURSUIT score, the GRACE score is determined to a large extent by the age of the patient, an element that holds only indirect evidence of coronary artery disease. Unfortunately, the GRACE investigators do not divide patients into different risk groups, making it less easy for the physician to interpret a patient’s individual score.

The FRISC score is quite comparable to the TIMI score, with a c-statistic of 0.70 for the combined endpoint, indicating only moderate discriminative power. The FRISC score is simple to calculate, but again allows only binary choices.

Furthermore, none of the scores emphasizes the value of patient history, despite the fact that clinicians rely heavily on this aspect. The clinical judgement of the treating physician will already divide patients into low, intermediate and high risk groups for an adverse event. Without doubt, clinicians have developed strong competence in patient selection, not requiring complex algorithms and computer based calculating tools. Therefore, the ideal risk score closely follows this clinical reasoning. Based on general impression, patient history, ECG characteristics, risk factors for coronary atherosclerosis and levels of cardiac markers, a quick estimation can be made of the individual patient’s risk. 

### Newly Developed Risk Score

The HEART risk score was specifically developed for chest pain patients presenting to the ED. The HEART score encloses each of the previous mentioned parameters of clinical judgement: History, ECG, Age, Risk factors and Troponin levels. The HEART score translates the clinical judgement into a uniformly comprehensive number of 0-10.

**Fig. (1):**
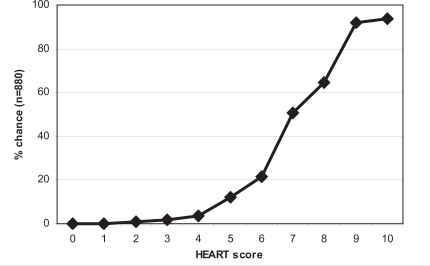
Probability of reaching a MACE in each HEART category [14].

Using the HEART score as guidance in the treatment of chest pain patients will clearly result in benefits for patients on both sides of the spectrum. The risk of MACE in patients with a HEART score ≤3 is 0.9%, 12% in patients with HEART score 4-6 and 65% in patients with a HEART score ≥ 7 (Fig. **1**) [14]. 

Well known markers of increased risk, such as higher age, presence of risk factors and history of coronary atherosclerosis, are all incorporated in the HEART score. The combination of the five elements will allow for a more firmly based decision, mainly in cases of atypical presentation or absence of ECG abnormalities. 

Compared with other risk scores, the HEART score is superior in terms of both simplicity and predictive power, not only for patients at high risk but also those patients at low risk for ACS (Table **VI**). Therefore it is quite useful for bedside clinical practice.

## CONCLUSION

Previously developed risk scores for chest pain patients are designed to identify the subgroup of ACS patients in the CCU who are at the highest risk of an adverse event. Most of the described risk scores were developed after identification of those risk factors which were independently associated with the primary endpoint, usually death and/or MI. Statistically, these scores have a firm basis. However, the selection of parameters and their individual weighting make them less applicable in the bedside setting. The recently developed HEART score for chest pain patients in the ED closely follows clinical reasoning. Therefore, it is far more applicable to the whole range of chest pain in the emergency setting. The HEART score appears a strong predictor of event free survival on one hand and potentially life threatening cardiac events on the other hand. A direct comparison of the various risk scores within one clinical study is desirable. 

## Figures and Tables

**Table I:** Composition of the PURSUIT Risk Score for Unstable Angina

Age (decade)	50	8
	60	9
	70	11
	80	12
Sex	Male	1
	Female	0
Worst CCS class past 6 weeks	No angina/CCS I/II	0
	CCS III/IV	2
Signs of heart failure		2
ST depression on ECG		1
**Total**		

**Table II:** Composition of the TIMI Score for Unstable Angina / Non-ST-segment Elevation Myocardial Infarction (NSTEMI)

**Historical**	
Age ≥ 65 years	0
	1
≥ 3 risk factors for CAD	0
	1
Known CAD (stenosis ≥ 50%)	0
	1
ASA use in past 7 days	0
	1
**Presentation**	
Recent (≤ 24H) severe angina	0
	1
↑ cardiac markers	0
	1
ST deviation ≥ 0.5 mm	0
	1
**Total**	

CAD = Coronary artery disease, ASA = Acetyl Salicylic Acid

**Table III:** Composition of the GRACE Score (2003)

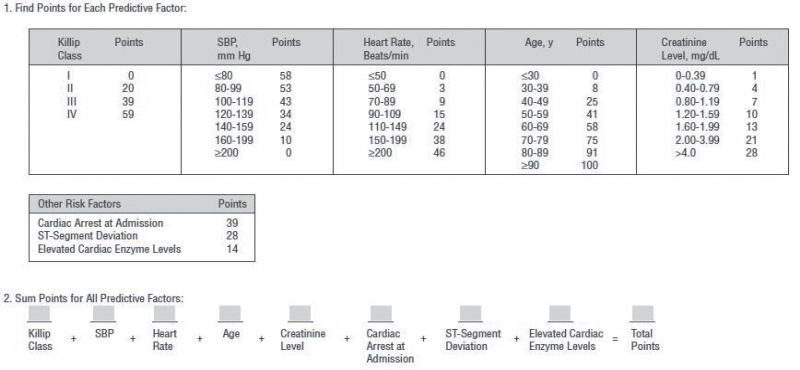

**Table IV:** Composition of the FRISC Risk Score for Unstable Angina

Age ≥ 70 years	0
	1
Male sex	0
	1
Diabetes	0
	1
Previous MI	0
	1
ST depression on ECG	0
	1
Elevated Troponin levels	0
	1
Elevated Interleukin 6 or CRP	0
	1
**Total**	

**Table V:** Composition of the HEART Score for Chest Pain Patients in the Emergency Department.

HEART Score for Chest Pain Patients		
**History**	Highly suspicious	2
	Moderately suspicious	1
	Slightly suspicious	0
**ECG**	Significant ST-depression	2
	Non specific repolarisation disturbance	1
	Normal	0
**Age**	≥ 65 year	2
	45 – 65 year	1
	≤ 45 year	0
**Riskfactors**	≥ 3 risk factors or history of atherosclerotic disease	2
	1 or 2 risk factors	1
	No risk factors known	0
**Troponin**	≥ 3x normal limit	2
	1-3x normal limit	1
	≤ normal limit	0
		**Total**

**Table VI:** Summary of Clinical Risk Scores for ACS, Modified After Morrow [15].

	PURSUIT		TIMI	GRACE	FRISC		HEART

Population	UA/NSTEMI		UA/NSTEMI	All ACS	UA/NSTEMI		All Chest Pain

Outcome	Death	Death/MI			Death	Death/MI	

**Key elements**	5		7	8	7		5
Age	X		X	X	X		X
Gender	X				X		
Prior MI/CAD			X		X		X
DM, CRF’s			X		X		X
Symptoms/History	X		X				X
Use of aspirin			X				
Weight							
HR				X			
SBP				X			
CHF/Killip class	X			X			
ECG	X		X	X	X		X
CKMB/cTn			X	X	X		X
Serum Cr				X			
Serum Interl-6/CRP				X	X		
Cardiac Arrest							

**Possible max score**	18		7	372	7		10

**c-statistic**	0.84	0.67	0.65	0.83	0.77	0.70	0.90

**Computer needed**				Yes			

PURSUIT = Platelet glycoprotein IIb/IIIa in Unstable angina: Receptor Suppression Using Integrilin (eptifibatide) Therapy, TIMI = Thrombolysis in Myocardial Infarction, GRACE = Global Registry of Acute Coronary Events, FRISC = Fast Revascularisation in Instability in Coronary disease, HEART = History ECG Age Risk Factors Troponin, UA = Unstable Angina, NSTEMI = Non-ST Elevation Myocardial Infarction, ACS = Acute Coronary Syndrome MI = Myocardial Infarction, CAD = Coronary Artery Disease, DM = Diabetes Mellitus, CRF’s = Cardiac Risk Factors, HR = Heart Rate, SBP = Systolic Blood Pressure, CHF = Congestive Heart Failure, ECG = Electrocardiogram, CKMB = Creatininfosfokinase MB, cTn = cardiac Troponin, Serum Cr = Serum Creatinin, Serum Interl-6 = Serum Interleukin-6, CRP = C-Reactive Protein.
